# Tailoring the Structure of Lipids, Oleogels and Fat Replacers by Different Approaches for Solving the *Trans*-Fat Issue—A Review

**DOI:** 10.3390/foods10061376

**Published:** 2021-06-14

**Authors:** Mishela Temkov, Vlad Mureșan

**Affiliations:** 1 Department of Food Technology and Biotechnology, Faculty of Technology and Metallurgy, Ss. Cyril and Methodius University in Skopje, Rudjer Boskovic 16, 1000 Skopje, North Macedonia; 2 Department of Food Engineering, Faculty of Food Science and Technology, University of Agricultural Sciences and Veterinary Medicine Cluj Napoca, 3-5 Manăștur st., 400372 Cluj Napoca, Romania

**Keywords:** *trans*-fat, oleogels, fat replacers, fat enzymatic modification, lipid structuring

## Abstract

The issue of the adverse effects of *trans*-fatty acids has become more transparent in recent years due to researched evidence of their link with coronary diseases, obesity or type 2 diabetes. Apart from conventional techniques for lipid structuring, novel nonconventional approaches for the same matter, such as enzymatic interesterification, genetic modification, oleogelation or using components from nonlipid origins such as fat replacers have been proposed, leading to a product with a healthier nutritional profile (low in saturated fats, zero *trans* fats and high in polyunsaturated fats). However, replacing conventional fat with a structured lipid or with a fat mimetic can alternate some of the technological operations or the food quality impeding consumers’ acceptance. In this review, we summarize the research of the different existing methods (including conventional and nonconventional) for tailoring lipids in order to give a concise and critical overview in the field. Specifically, raw materials, methods for their production and the potential of food application, together with the properties of new product formulations, have been discussed. Future perspectives, such as the possibility of bioengineering approaches and the valorization of industrial side streams in the framework of Green Production and Circular Economy in the production of tailored lipids, have been highlighted. Additionally, a schematic diagram classifying conventional and nonconventional techniques is proposed based on the processing steps included in tailored lipid production as a convenient and straightforward tool for research and industry searching for healthy, sustainable and zero *trans* edible lipid system alternatives.

## 1. Introduction

Natural fats and oils have unique physicochemical, nutritional and rheological characteristics determined by their triacylglycerol (TAG) composition [1]. Solid fats usually contain higher proportions of saturated fats, whereas liquid oils are richer in mono- and polyunsaturated fats [2]. Existing forms of fats are not always suitable for their intended purposes in industrial applications. Saturated fats exhibit several functional properties that many food technologies and products are based on. For example, fat shortenings prevent gluten network formation in baked goods by coating gluten particles due to their plasticity and elasticity or forming sheet layers in croissant dough. Moreover, solid fats are being used as spreadable products because of their unique property to experience a plastic flow when a stress higher than the yield stress is applied [3,4]. Most of these functional properties and mechanical characteristics belong to animal fats or palm oil. Another problem that needs to be addressed is the sustainability of the sources caused by the widespread deforestation and environmental loss of palm trees around the world [3].

Therefore, in order to replace the existing solid fats with liquid oil, modifications to the latter group must be made for functionality improvements. Over the years, numerous approaches has been developed for oil modification. The conventional techniques include: hydrogenation, fractionation or chemical interesterification [5,6,7]. These modified fats are often referred as structured or designed lipids [1]. Partial hydrogenation forms *trans*-fatty acids, which are linked with negative effects on human health by increasing the levels of “bad cholesterol” and complementary decreasing the levels of “good cholesterol” [4]. Moreover, since the *trans*-fatty acids intake is limited to 2 g/100 g by the European Commission, starting from 24 April 2019 by Regulation No. 649 [8], researchers and food producers have developed alternative paths in obtaining lipids with “zero *trans*-fatty acids”, yet with the desired rheological characteristics.

One of the unconventional ways is the enzymatic interesterification that has many advantages over chemical interesterification, such as: mild processing conditions, less purification steps and less energy consumption and, thus, is more economical [9]. In this process, the enzyme lipase trims the ester bonds between glycerol and fatty acids randomly, after which, the free fatty acids (FFA) are reattached on a different position of the same molecule of glycerol (intraesterification) or on a different molecule of glycerol (interesterification) [10].

Interesterification comprises three types: (a) acydolysis (reaction between FFA and TAG), (b) alcoholysis (reaction between alcohol and TAG), as, for example (b’) glycerolysis (reaction between glycerol and TAG) and (c) transesterification (splitting FA from glycerol and re-esterifying them on different sn-positions) [1]. This type of lipid structuring was applied on plastic fats [11], human milk substitutes [12], structured lipids enriched with essential oils [13], cocoa butter equivalents [14] and low-calorie structured lipids [15]. Nicholson and Marangoni [16] went one step further and proposed an innovative conversion of the liquid oil into a solid by enzymatic glycerolysis to increase the crystallization temperature without changing the fatty acid composition. In a recent work, lipase-catalyzed glycerolysis was implemented to structure cottonseed and peanut oil [17]. This structured lipid has the required solid fat content (SFC), as well as the nutritional benefits. Moreover, Wozniak at al. designed the enzymatic reaction of interesterification towards hydrolysis and used the produced byproducts such as monoacylglycerol (MAG) and diacylglycerol (DAG) as emulsifiers to obtain a stable oil-in-water emulsion [18]. An extensive review of the literature of the last 5 years related to enzymatic interesterification of fats, its mechanisms, research and their commercial application was reported by Sivakanthan and Madhujith [1].

Another relatively new unconventional strategy to the alternative of “zero *trans* fats” is oleogelation, which represents transforming the liquid oil into “gel-like” viscoelastic material without changing its chemical structure [19,20]. For this purpose, different organogelators are used. The two major groups of oleogelators include (1) low molecular weight oil gelators (LMOG) able to self-assemble and induce crystallization of the oil phase and (2) high molecular weight oil gelators (HMOG) able to organize themselves into a 3D network, which can trap a large amount of oil into a gel-like structure [21]. The first group consist of: waxes, MAG, DAG, fatty acids, alcohols, ceramides, phospholipids and phytosterols. Despite their low molecular weight, tiny concentrations in the order of 0.5% *w/w* are sufficient to form a gel. Organogels made with LMOG are thermoreversible and shear-sensitive due to the noncovalent interactions [4]. The second group comprises, as the name suggests, polysaccharides and proteins with high molecular weights, such as: ethyl cellulose, methyl cellulose, hydroxypropyl methyl cellulose, chitin and chitosan, gelatin, hydrophilic and hydrophobic proteins [22]. These oleogels pose viscoelastic properties dependent on the type of the gelator, its molecular weight and concentration [4]. For a substance to be efficient as an organogelator, it should have a solubility in the solvent (oil) somewhere in between high soluble and insoluble. High soluble substances will form solutions instead of gels, whereas insoluble ones will precipitate without even interacting with the solvent. Several basic methods have been developed in oleogel preparation, namely: (a) direct dispersion of oleogelators in the liquid phase, (b) an indirect method using water continuous emulsions as templates, (c) structuring aided by physical sorption of liquid oil and (d) structuring using biphasic systems. The direct dispersion method usually involves lipid-based gelators that are melted in the oil phase and subsequently cooled down to room temperature, followed by nuclei formation, crystal growth and network formation. The indirect method using water-continuous emulsion is based on the adsorption of a hydrophilic biopolymer on the water–oil interface, with a subsequent removal of the water to achieve a structure with entrapped oil inside. The method for structuring the oil using the sorption process is based on the enrichment of the material on its interface using porous compounds with high specific contact areas (aerogels, hydrogels and cryogels). The structuring of fats using biphasic systems can be categorized as (a) water-continuous emulsions structured by biopolymers, (b) highly concentrated water-continuous emulsion structured by the close proximity of the droplets and (c) oil-continuous emulsions structured by fat particles in a crystal network [19]. The potential of the future production of oleogels can be found in bioprocessing fermentation technologies and the use of oleogelators from renewable sources such as food processing side streams. In the framework of Circular Economy, Papadaki et al. developed an oleogel using a microbial wax ester as a substitution of a conventional wax ester. This research group used side streams: sugarcane molasses from the sugar extraction process and soybean cake from the soybean oil extraction process for the fermentation and production of microbial oil, which was enzymatically transformed into wax ester and subsequently used as an oleogelator [23].

An additional alternative group that contributes to saturated fat reduction in food and less fat intake is fat replacers. This group can be partitioned into two sections, namely: fat substitutes and fat mimetics. Fat substitutes are structured lipids that have the physical and functional properties of conventional lipids and are suitable for different technological operations. In addition, fat substitutes provide reduced or no calories at all and can replace conventional lipids in a proportion of 1:1. On the other hand, fat mimetics are generally polar, water-soluble compounds (protein or carbohydrate-based) that can mimic some of the sensorial or physical properties of conventional fats. They cannot fully replace conventional food lipids, and moreover, they are not appropriate for certain technological operations. However, their high water-binding capacity creates the desired sense of creaminess and mouthfeels in foods comparable to the texture of a full-fat product [24]. This field is under extensive research worldwide. In the past year, fat has been successfully replaced in several food products: meatballs [25], mayonnaise [26], filling cream for sandwich cookies [27], fermented sausages [28,29], and muffins [30].

Categorizing the lipid-structuring approaches is still a matter of discussion between researchers. For example, Patel et al. [3] reported a schematic diagram where all structured lipids are called fat mimetics and the methods for structuring are divided into three major groups: indirect oil structuring via a solvent exchange and colloid templates, oleogelation using lipidic and nonlipidic gelators and structured biphasic systems. On the other hand, O’Connor and O’Brien [24] categorized fat replacers into two major groups, namely as fat substitutes (full and direct replacements of conventional lipids) and fat mimetics (mimics some of the lipid properties but cannot fully replace conventional lipids). Furthermore, structured oils were categorized as interesterified lipids, oil bulking systems, organogels and structured emulsions that were subdivided into hydrogel oil-in-water (O/W) and water-in-oil (W/O) emulsions or organogel O/W and W/O emulsions [31].

**Figure 1:**
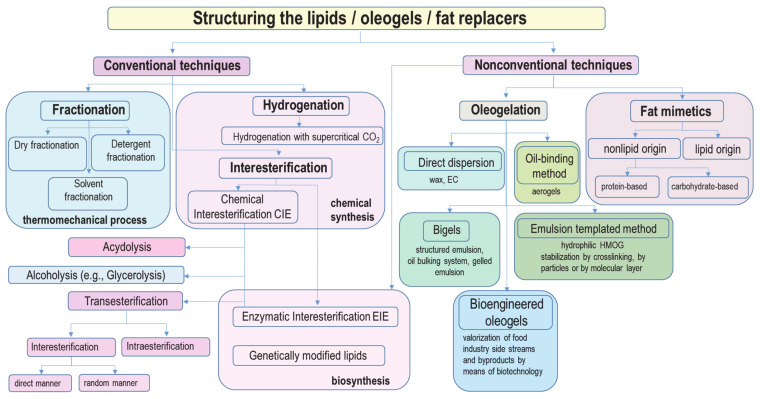
Schematic diagram for various approaches implemented in edible lipid structuring.

The objective of this review is to critically discuss all the available tailoring techniques for lipids, oleogels and fat replacer structuring for solving the *trans*-fat issues in food technology. Moreover, a schematic diagram classifying the conventional and nonconventional approaches is proposed (Figure 1) as an easy-to-use tool for research and industry searching for healthy, sustainable and zero *trans* edible lipid system alternatives.

## 2. Conventional Structured Lipids

### 2.1. Fractionation

Fractionation is widely used conventional technique for lipid structuring and their chemical and physical property alterations for better performance and suitability in various applications. While hydrogenation and chemical interesterification cause irreversible chemical changes in the lipid composition, fractionation is a thermomechanical reversible process. The main principle of the fractionation of fats is the separation of the existing TAG with different melting points (depending on the molecular weight and the degree of unsaturation) in two different products (stearin and olein), which can be further processed. There are three types of fractionation: dry fractionation, solvent fractionation and detergent fractionation.

In the process of dry fractionation, the crystals are formed from the melt without the addition of any additive. Solvent fractionation involves the use of solvents such as acetone or hexane and is based on the different solubilities of TAG at a given temperature. The process of detergent fractionation starts with dry fractionation, after which a surfactant aqueous solution is added to relocate the crystals from oil phase to aqueous phase and assist following the separation [32]. The fractionation process was commercialized several decades ago and used for obtaining palm oil fractions to be used in all kind of different products (stable creams, sauces, infant formulas, ice cream, bakery products and chocolate) [33]. However, the process has received more attention with the concerning effects of *trans*-fatty acids, due to its simple and cheap technique. Dry fractionation was improved with introducing multistep crystallizations separated with membrane press filters allowing the manufacturing of fractions for different applications, such as top oleins, palm red fractions and palm mid fractions (solvent-free cocoa butter equivalent; CBE) [33].

By controlling the oil/solvent ratio and temperature of fractionation, CBE with different melting temperatures can be obtained from high stearic–high oleic sunflower oil in a single-step process [34]. Cocoa butter improver (CBI) is used in blends together with cocoa butter for manufacturing heat-resistant chocolate products for tropical climates. It is usually made from high-melting symmetrical monounsaturated TAG. Jin et al. [35] reported CBI production from mango (*Mangifera indica* L) kernel fat in three-staged acetone fractionation, where the second fraction showed the highest high-melting symmetrical monounsaturated TAG content (94%). On the other hand, when a similar work was done by the same research group with a different solvent (high-purity isohexane containing 88.12% 2-methylpentane), the third stearin contained only 69.2% symmetrical monounsaturated TAG [36]. Moreover, symmetric monounsaturated TAG with a high yield and free of tripalmitin were produced from the second fractionation in two-stage acetone fractionation from palm stearin as the starting material [37]. The research is also ongoing in the directions of amelioration of the process and understanding the effects of the processing conditions. In that sense, cooling rates, end-crystallization temperatures and agitation speeds caused significant changes in the melting and crystallization behaviors of the olein fraction of palm-based diacylglycerol but did not affect the stearin fraction [38]. Combining processes is a useful tool for tailor-made lipids. Accordingly, soft stearins were produced from high oleic–high stearic (HOHS) sunflower oil by dry fractionation, which were further processed by solvent fractionation for high-melting sunflower stearin production. The choice of solvent and oil/solvent ratio can affect the crystallization rate, melting profile and fractionation temperature [39]. The concerning effect of the solvent usage that needs to be removed and the fat refined after the process can be overcome by simultaneous supercritical CO_2_ fractionation, where refining is not needed. The first fraction is solid due to its highest solubility in supercritical CO_2_, followed by semiliquid and liquid fractions. At a lower pressure, the palm kernel oil solubility decreases in supercritical CO_2_ along with the temperature and vice versa. The first fraction was rich in short-chain TAG, while the second fraction had long-chain and unsaturated TAG in large quantities [40]. Besides many advantages that this modification process has, there are several drawbacks, such as high energy and material costs, safety issues, consumer acceptance, solvent recovery, low separation efficiency or the palm and other tropical fat sustainability.

### 2.2. Chemical Synthesis

**Figure 2:**
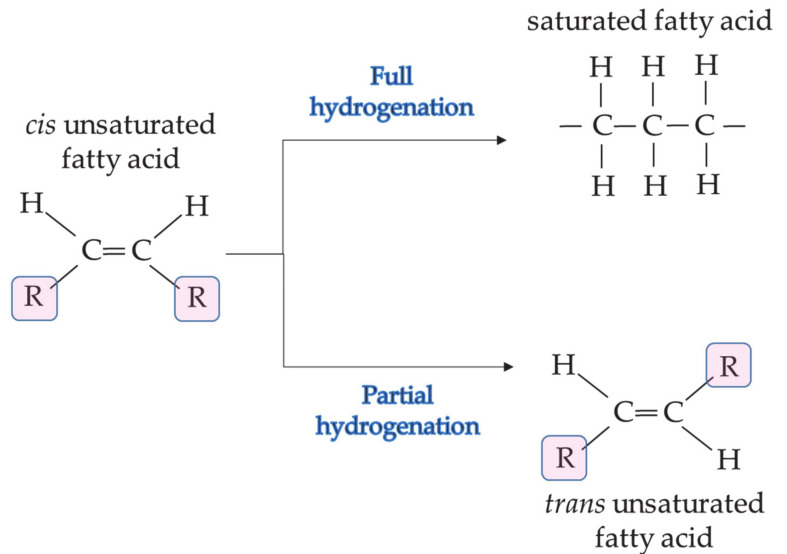
Schematic representation of full and partial hydrogenation.

Besides fractionation, hydrogenation and chemical interesterification are well-established industrial processes for lipid structuring. Hydrogenation was discovered at the beginning of the last century and soon after became the revolution in oilseed industry, as it made liquid oils transform into the appropriate consistency and be used in margarines, shortenings and creams. The simple principle of hydrogenation is the addition of a hydrogen atom to the unsaturated bonds in a presence of a catalyst, leading to an oxidative stable product with characteristics of solid materials (Figure 2). The process is controlled by the temperature, pressure, rate of agitation, amount of the catalyst and purity of the hydrogen. Partially or fully hydrogenated fat should undergo purification and bleaching processes after manufacturing. During the process, some of the double bonds are saturated and some relocated to a new position in a *trans* form [41]. These *trans* fats are associated with coronary heart diseases and blood regulation flow by the calcification of arterial cells and inhibition of the enzyme cyclooxygenase that converts arachidonic acid into prostacyclin, respectively [42].

In industrial hydrogenation, the content of these harmful acids may reach up to 60% [43]. A comprehensive review on heterogeneous hydrogenation focused on the kinetic rate expressions, selectivity and the production of *trans*-fatty acids under different conditions, including mass transfer limitations and intraparticle transport, was reported by Veldsink et al. [44]. A recent chapter explaining the principles of the hydrogenation reaction, factors affecting the reaction and their combination was published by Patterson [5]. The content of *trans*-fatty acids can be reduced if the hydrogen concentration is increased; nevertheless, the hydrogen low solubility in oil and mass resistance in the interface limit the aforementioned technique. Therefore, Harrod and Moiler [45] solved the problem with the solubility by adding propane to the reaction mixture and converting the mixture in its supercritical state. With these modifications in the process, they achieved a higher (up to 1000 times) productivity and decreased concentration of *trans*-fatty acids while using the same consumption of the catalyst.

**Figure 3:**
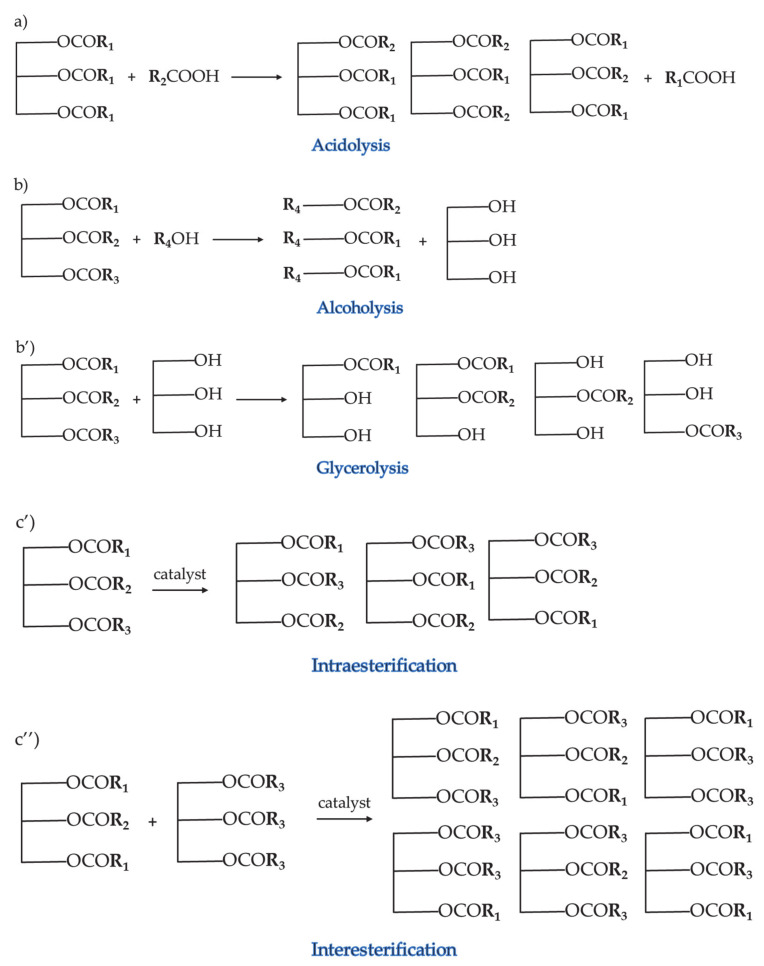
Schematic representation of interesterification (chemical or enzymatic): (**a**) acydolysis, (**b**) alcoholysis, (**b’**) glycerolysis, (**c’**) transesterification—intraesterification and (**c’’**) transesterification—interesterification.

As the hydrogenation with propane causes the production of large amounts of n-alkanes as byproducts, this technology was developed using supercritical CO_2_. This nonflammable and nontoxic gas is miscible with H_2_ and has a low critical temperature and pressure (31 °C and 7.4 MPa, respectively), which allows hydrogenation to be accomplished in less severe conditions [46,47,48]. Moreover, this type of hydrogenation can occur in one-phase systems or binary systems, the first one being more advantegeous but limited due to the low solubility of oil supercritical fluids [48]. In addition, the solubility of CO_2_ was ameliorated in electrolytes, which gave better optimal conditions for the electrochemical hydrogenation of soybean oil [46]. Lipid hydrogenation using critical CO_2_ was comprehensively reviewed by Wang, Liu and Bao [49]. Furthermore, different techniques of hydrogenation: electrocatalytic hydrogenation, precious catalyst hydrogenation and supercritical fluid hydrogenation, as well as the hydrogenation technique for the high accumulation of conjugated linoleic acids, were reviewed by Jang et al. [47]. Since the industrialization of hydrogenation with supercritical CO_2_ is not economical, and conventional partial hydrogenation produces lot of *trans*-fatty acids, other alternatives for oil structuring over the years have been developed. Other supercritical fluids were used as reaction solvents for vegetable fat hydrogenation [50]. Chemical interesterification represents an industrial alternative to hydrogenation for lipid physical property improvements in the food sector and involves a catalyst to attain the goal of redistribution of the fatty acids on the glycerol backbone. As it was mentioned before, interesterification can be divided into several types of reactions: acydolysis, alcoholysis/glycerolysis and transesterification, with the latter being divided into intraesterification (redistribution of FA within the same TAG) and interesterification (redistribution of FA on another glycerol from a different TAG) (Figure 3). The process starts with activation of the catalyst, followed by trimming the ester bonds to obtain fatty acids and glycerol and then reshuffling the fatty acids on a different position. The catalyst (alkylates of sodium and sodium/potassium alloys) enables the interesterification to be performed at lower temperatures, avoiding the reaction of polymerization and decomposition to occur [51]. Moisture, free fatty acids and peroxides in the oil should be kept under 0.01% (*w*/*w*) and 0.1% (*w*/*w*), respectively, as they weaken the catalyst’s performance. The reaction is terminated by the addition of water and diluted acid, followed by removing the catalyst, with a substantial fat loss. Interesterification can be accomplished in a random or direct manner, where the reactions occur at temperatures above or below the melting point of the highest melting TAG in the blend. By the first method, fatty acids interchange freely among TAG molecules until the equilibrium is reached, whereas, in the second method, saturated TAGs are eliminated from the mixture with the equilibrium directed at crystallizing more of them [2]. Chemical interesterification nowadays presents the research interests for the vast scientific community. For example, Soares et al. [7] used palm stearin, coconut oil and canola oil and Fauzi, Rashid and Omar [52] employed palm stearin, palm kernel oil and soybean oil to produce *trans*-free soft margarines with a reduced SFC, consistency and melting point by chemical interesterification with sodium methoxide as the catalyst. In addition, Farfán et al. [53] interesterified a binary blend of fully hydrogenated soybean oil and walnut oil in ratios of 20:80, 40:60 and 60:40 *w*/*w* to produce zero *trans*-fatty acid shortening with decreased SFC and improved nutritional value. Motamedzadegan et al. [54] and Naeli, Farmani and Zargaraan [55] studied the effects of chemical synthesis of the blends of virgin coconut oil with palm olein and palm stearin with soybean oil, respectively, in ratios of 10:90, 20:80, 30:70, 40:60 and 50:50 *w*/*w*. The blends prior the esterification were not suitable for practical application due to a high melting point and low elasticity, whereas chemical processing introduced those attributes to the blend but, on the other hand, increased the content of FFA. In addition, the obtained structured lipids were free of *trans*-fatty acids and displayed lower storage (G′) and loss (G″) moduli. Chemical interesterification improved the melting point, consistency and solid fat content at a refrigeration temperature, as well as the balance between saturated and unsaturated FA in a blend composed of patawa oil and palm stearin in the ratio 50:50 [56]. Ribeiro et al. [57] prepared structured lipids from high oleic sunflower oil and fully hydrogenated soybean oil in a ratio of 50:50 *w*/*w* by chemical and enzymatic interesterification and reported the benefits of biosynthesis over the chemical synthesis, such as regiospecificity and lower acyl migration. In a recent study, nanocrystalline calcium oxide was used as a catalyst in the chemical structuring of a blend composed of soybean oil and methyl acetate in the molar ratio 1:40 [58]. For more profound theoretical knowledge and reaction mechanisms, the effects of chemical interesterification on the physical and chemical properties of lipids and their applications in various food products, refer to Rousseau et al. [51] and Gibon and Kellens [59].

### 2.3. Biosynthesis (Enzymatic Interesterification)

The interest in enzymatic interesterification (EIE) of fats and its application in food matrices has risen significantly over the past three years. Many scientists have dedicated their research to creating products that will be beneficial in the protection of human health. The application of various EIE fats in different food products is summarized in Table 1. In the introduction, the health benefits of reduced *trans*-fatty acids was already discussed. In addition to this, Santos et al. [60] used enzymatically esterified blends composed of milk fat and palm olein enriched with probiotic bacteria *Bifidobacterium animalis* subsp. *lactis* Bb-12. The melting point of the blend was decreased, whereas the fats being part of the blend showed eutectic softening. Other researchers reported better rheological behavior of the interesterified fats. In this sense, the EIE blend of hybrid palm stearin and palm kernel oil gave a better performance in terms of the melting and crystallization temperatures and faster structuring, as well as a reduction of the size of the crystals preventing graining [61].

Pork jowl was replaced with an enzymatically interesterified blend of milk fat and rapeseed oil in the proportion of 3:2 *w*/*w*, where no loss of texture and thermal drip was reported. Additionally, a higher content of linoleic acid was shown in the same research [63]. Frying shortening was ameliorated by enzymatically interesterifying palm stearin and canola oil with immobilized and nonimmobilized lipase in various supports. The novel product had zero *trans*-structured lipids, which can be used for higher temperature applications. Cocoa butter is one of the fats that has a unique narrow melting profile due to the TAG composition and requires tempering for crystallization in β′ polymorph for a smooth texture. The chocolate should be hard at room temperatures with a good snap but should start melting at the temperature of the mouth. Substitute fats generated from lauric fats are considered the only effective substitution fats. An attempt was made to produce a cocoa butter substitute using a mixture of palm kernel oil and interesterified palm olein, fully hydrogenated palm oil and palm kernel oil. The mixture showed eutectic effects in the temperature range of 15–35 °C, while the crystallization occurred predominantly in β′ form. The produced chocolate had good hardness and fracturability [64]. In addition, the cocoa butter substitute was prepared from a mixture of *Cinnamomum camphora* seed oil and fully hydrogenated palm oil by enzymatic interesterification in a solvent-free system. The saturated fats in the esterified blend were reduced, and the novel TAG were composed mainly of capric and lauric acid. The interesterified blend had a melting point similar to a commercial cocoa butter substitute [14]. By enzymatic interesterification, a substitute for human milk was obtained containing 76% palmitic acid at the sn-2 position, 0.3% arachidonic acid, 3.4% eicosapentaenoic acid and 4.25% docosahexaenoic acid with a melting point of 42 °C [12]. Another research group produced a human milk fat substitute from a base of catfish oil and coconut oil via interesterification. They reported a product with a TAG composition similar to human milk fat rich with medium and long-chain triacylglycerol (62%) with 16:0 FA in the sn-2 position and long-chain fatty acids (39%) [65]. In a recent study, two different blends (palm stearin (PS)/soybean oil (SO); PS/SO and palm stearin/rapeseed oil (RS); PS/RO) were interesterified for the production of fast-frozen special fat, and their storage stability over a period of 4 weeks was evaluated. Both blends exhibited good oxidation stability, whereas the SFC and hardness decreased with increasing the temperature. The PS/RO blend had a slower transformation from β′-polymorph to β-polymorph, indicating a better quality over an extended period of storage [67].

#### Structuring Vegetable Oil by Enzymatic Glycerolysis

Enzymatic interesterification usually refers to transesterification, whereas glycerolysis represents a part of interesterification used to covert the FFA into MAG, DAG or TAG. It is usually applied to decrease the content of FFA, so that transesterification can move forward. For this alternative esterification to occur, excessive contents of glycerol, high temperatures and a catalyst are needed, since the solubility of the glycerol in organic solvents is very low. Liberated water from the reaction should be removed. In the early phase of the reaction MAGs are formed, which subsequently decrease in the later phase due to their conversion to DAGs and TAGs [69].

This alternative enzymatic interesterification was applied in an innovative technique for the direct conversion of liquid oil into solid fat conducted by Nicholson and Marangoni [16]. Namely, TAGs were lipolyzed to MAG and DAG, which served as alternators of the crystallization temperature without changing the fatty acid composition, yet still having the solidity required for different functions. The fats produced by the described mean were used as a substitute for palm oil and other saturated fats as their sustainable alternative. There were additional studies providing the health benefits of DAGs, such as reducing LDL and total cholesterol due to the presence of the 1,3-DAG isoform in the DAG mixture. Nicholson and Marangoni [16], in their study, used cottonseed oil and immobilized lipase B extracted from *Candida antarctica* as a biocatalyst. They observed MAG and DAG rapidly increasing from 1% to over 20% and from 7% to 43%, respectively, within 18 h of the reaction. At the end of the reaction (72 h), the total content of the MAGs was 34.3% and 49.1% for the DAGs. By changing the molar concentration of glycerol in the reaction mixture from 0.5 to 1, the MAGs content was higher, whereas the DAGs content was not affected. The SFC of cottonseed oil increased over time, i.e., from 8.2% to 14.6% after 6 h to 22.4% after 24 h and to 26.2% after 48 h, at which point, the maximum structuring potential was observed. Using different molar ratios of glycerol:TAG (0.10:1, 0.25:1, 0.5:1, 1:1, 2:1 and 4:1) the researchers observed different SFC at 5 °C, such as 15.8%, 18.3%, 21.9%, 22.6%, 22.4% and 20.6%, respectively. Moreover, the SFC increased as a relation to the MAG/DAG content, reaching a plateau after 48 h. They concluded that, at higher temperatures, the crystalline material is determined by the MAGs content, whereas, at lower temperatures, the DAGs content influences the SFC. The higher crystallization temperature was confirmed with differential scanning calorimetry and was a result of the isomeric form of 1,3-DAGs that crystallize approximately 10 °C higher than TAG. The novel fat also showed an improved oil-binding capacity, which enhanced as the reaction of glycerolysis proceeded from 17% for native cotton seed oil to 91% for the structured fat at the end of the reaction, which clearly showed the dependency of SFC. When the reaction was performed with a 1:1 glycerol:TAG molar ratio, no oil loss was observed. In terms of the microstructure, the fat produced from the 1:1 glycerol:TAG molar ratio had smaller crystals in larger numbers in greater homogeneity in comparison to the fats obtained from the 0.5:1 and 2:1 glycerol:TAG molar ratios. These results were confirmed with the isothermal crystallization experiment, where the fat obtained from the 1:1 glycerol:TAG molar ratio demonstrated the fastest crystallization kinetics, leading to a firmer product. The esterified product was applied in the manufacturing of soft-tub margarine, demonstrating a flow behavior and hardness similar to the commercial ones [16]. Moreover, cottonseed oil, peanut oil, soybean oil, rice bran oil, olive oil, sesame oil, canola oil, HOAO and tigernut oil were converted into structural fats by lipase-catalyzed glycerolysis in a very recent research conveyed by Nicholson and Marangoni [17]. Glycerolysis was performed using 2 *w*/*w*% non-regiospecific *Candida antarctica* lipase B, immobilized on Immobead 150 at 65 °C for 48 h at the glycerol:TAG molar ratio 1:1. Under these conditions, after, the glycerolysis oil was composed approximately of 30% MAGs, 40–50% DAGs, and 20% of residual TAGs, regardless of the type of oil. The crystallization onset temperature was increased by approximately 20 °C due to the presence of MAGs and DAGs and was also affected by the presence of the content of saturated fatty acids in the system. Most of the structured oils had similar needle-like crystals of 50–100-μm diameters, except tigernut oil, peanut oil and olive oil, which contained a microstructure of pallet-like crystals of 20–50 μm tending to aggregate. They concluded that the microstructure of the systems was influenced by the presence of monopalmitin in MAG and the absence of DAG. In all the studied oils, SFC was increased in the treated samples compared to the untreated measured at 5 °C, except the HOAO glycerolysis product, which did not contain any solids at the given temperature. It was found that the SFC content was influenced by the combination of the ratio of oleic–linoleic acids present in the MAGs and DAGs, as well as by the content of saturated fatty acids, which was confirmed to be at least 10%. When the structuring is influenced only by the saturated fatty acids, and there is a little or no contribution from oleic acid, the melting profile is very gradual, which gives a negative impact on the sensory perception, as it leaves many solids at the mouth temperature. Among all studied oils, the tigernut glycerolysis product had the highest SFC at 5 °C and showed a good potential to be used in soft-tub margarine and fat spreads, showing minimal phase separation after 10 months of storage and a forced deformation profile similar to commercial margarine. Moreover, the product showed a good melting profile, with only 3.2% solids remaining at 30 °C and 0% remaining solids at 35 °C.

In a separate research, Subroto et al. [68] synthetized structured lipids containing high amounts of MAG and DAG from a palm stearin–olein (PS-PO) blend through a combination of enzymatic glycerolysis and enzymatic interesterification in one system. The biocatalyst used for this reaction was immobilized lipase from *Candida antarctica*. The amount of solvent greatly affected the reaction rate, i.e., a higher solvent:fat ratio (2:1) increased the production of MAG (1.78 times) and TAG (1.25 times) due to the lower viscosity, followed by higher mass transfer of the reactants. In the same trend, as in a previously discussed study, the higher conversion rates of MAG (1.33 times) and TAG (1.25 times) were reached when the glycerol:fat ratio was increased from 1:1 to 1.5:1, whereas an increase of the 2:1 glycerol:fat ratio did not affect the MAG, DAG, or TAG conversion rates. Moreover, at a higher glycerol-to-fat ratio, the conversion rates decreased due to the higher viscosity. As the enzyme increased from 10% to 15%, the conversion of MAG, DAG and TAG increased 1.40, 1.29 and 1.29 times, respectively. The saturation level of MAG and DAG increased particularly with palmitic and stearic acid than the TAG in the structured lipid due to the specificity of the lipase towards the saturated acids. The structured lipid had a higher melting point and hardness compared to the liquid blend, which was expected because of the higher MAG and DAG contents. The emulsion capacity and stability of the structured fats were estimated as 60.2% and 96.8%, respectively.

### 2.4. Genetically Modified Lipids

The revolution in genetic engineering opened the door in structuring the lipids and altering the fatty acid profile directly into plant by identification, extraction, cloning and transferring targeted genes that will induce the synthesis of oil in a desirable, flexible manner. This modern use of oilseed biotechnology gave a completely new sight to production of structured lipids, mainly saturated in common crops, which will no further require catalytic hydrogenation to be stabilized. Most of the genes involved in lipid biosynthesis are being isolated and can be used in engineering techniques for tailor-made lipids. For example, synthesis of fatty acids in sunflower seeds occurs in the plastids and endoplasmic reticulum with participation of stearoyl-ACP desaturase, oleoyl-CoA desaturase and thioesterases. First enzyme transforms stearic to oleic acid by unsaturating at C9 in the fatty acid, second enzyme catalyzes the unsaturation at C12 converting the oleic into linoleic acid, whereas the third enzyme is involved in transportation of the of the synthetized fatty acids from the plastid to the endoplasmic reticulum [70]. By introducing sunflower mutants that express high stearic feature on high oleic background, high-stearic-high-oleic (HSHO) sunflower oil can be obtained, which can be further fractionated into stearins with matching physical properties of cocoa butter [71]. This type of genetically engineered sunflower oil had higher fraction of SFC, thus higher melting point, was more viscous and less prone to oxidation as well. Conventional peanut oil with high proportion of linoleic acid is prone to rancidity, off-flavors and short shelf-life. For broader application in food technology and better manufacturing of superior health-related products high oleic peanut oil is preferred [72]. Genetically engineering approaches by identification of different ahFAD2 gene families and its modification by modern molecular techniques (sequence-specific nucleases, zinc finger nucleases, transcription activator-like effector nucleases, clustered regularly interspaced short palindromic repeats etc.) for regulated gene expression for breeding high oleic peanut genotypes was extensively discussed in the review of Nawade et al. 2018 [73]. More precisely, suppression of ahFAD2B desaturase gene and mutation of ahFAD2A by insertion of miniature inverted-repeat transposable element induced creation of high-oleate phenotype of cultivated peanut [74,75]. Moreover, gamma rays mutagenesis induced new high-oleic mutant lines in oleoyl-PC desaturase in peanut that could serve as marker-assisted selection for variable fatty acid profile in future peanut breeding programs [76]. The two fatty acid desaturase genes (FAD2-1A and FAD2-1B) were genetically engineered by the transcription activator-like effector nucleases technique in soya bean that led to radical increase of the oleic acid from 20% to 80% and rapid decrease in linoleic acid from 50% to 4% [77]. High oleic safflower (up to 70%) was bred by profiling microRNA populations that differ in expressing high oleic and high linoleic genotypes [78]. Van Erp et al. achieved amazing work in biosynthesis of engineered human milk fat substitute in model oilseed *Arabidopsis thaliana*. [79]. What differs human milk from other fats is its unique stereoisomeric structure of TAG composed of palmitoyl (C16:0) at the sn-2 position, a task difficult to obtain with the existing methods of structuring. However, the group of van Erp et al. [79] modified the metabolic pathway for TAG biosynthesis by transferring the enzyme lysophosphatidic acid acyltransferase to the endoplasmic reticulum so it can attach C16:0 to the sn-2 position, thus increasing palmitoyl content by 20 times. Increasing the accumulation of saturated fatty acids by specific traits incorporation in plants, plant oil gains plastic-elastic features and becomes suitable for margarine and shortening applications [80]. However, genetic modification of plant storage lipids should not compromise the ability of the seed to grow and develop by interfering with the synthesis of plant structural lipids, neither to limit the crop yield, nor to induce cross-pollination with neighboring unmodified crops and affect biodiversity [80]. Additional limitations that have to be considered are the cost of raw materials, regulatory issues and the acceptance of the consumers [81].

## 3. Oleogels in Food

### 3.1. Conventional Oleogels

In the recent years there was a pronounced expansion in the research of oleogels and their potential as replacers of conventional solid fats. In industrial processing fats with higher SFC at room temperature are preferred due to their better oxidative stability hence better shelf life, good spreadability, the mouthful sense, the increased melting point and the snap. Nevertheless, when vegetable liquid oil is transformed into solid fat by the means of partial hydrogenation *trans* fats were created associated with coronary diseases. Even though saturated fats have positive functional attributes and play a key role in the processing of food, their excessive consumption may lead to many health disorders in the human body. The ideal fat should have the chemical composition of liquid oil (high fraction of mono and polyunsaturated fats) but all the functional properties of a solid fat. This idea of the novel product free of *trans*-fatty acids led to the original method of structuring called oleogelation. The attention is focused on developing methods and formulations of oleogels that will replace conventional fats in as many food products as possible without visual and drastic change in the product. Oleogels can be created by using crystalline particles or self-assembled low molecular weight compounds and self-assembled high molecular weight polymers. In the first method, liquid oil is trapped in self-assembled fibrous network, whereas in the second method the three-dimensional network is formed with the help of the polymers. In the formation process LMOG are heated to their melting temperature and mixed with oils, followed by cooling to room temperatures [22]. In case of hydrophilic HMOG, surfactant free oil-in-water emulsion is formed stabilized by the biopolymers, which is followed by removal of the water phase [82]. Type of oil and oleogelator contribute in the physical characteristics of the obtained oleogel. Waxes can structure lipids in low concentrations such as 0.5% (*w*/*w*) for rice bran wax used in solidifying rice bran oil [83]. On the other hand, Alvarez-Ramirez et al. [84] used candelilla wax at 5% (*w*/*w*) to structure canola oil that replaced the fat in sponge cakes. The cakes were more cohesive and softer, nonetheless the digestibility of starch was increased from 70% to 84%. Pork fat in meat pate was replaced by oleogel on linseed oil base structured by beeswax at 8% (*w*/*w*), obtaining a substitute product with increased polyunsaturated fatty acids, but decreased hardness and adhesiveness [85]. More compact and lighter Bologna sausages were also produced with sunflower based oleogel structured with monoglycerides with 50% of pork replacement [86]. Pork backfat in dry-cured sausages can be partially replaced (20% and 40% levels of replacement) by linseed based oleogels modified by beeswax and a mixture of γ-oryzanol and β-sitosterol but with adjustments in the drying process [87]. Different concentrations (3%, 5%, 7% and 9%, *w*/*w*) of rice bran wax were used in expeller-pressed (EP) corn germ oil oleogels, which were used as a full replacement of shortening in cookies without any significant difference compared to commercial cookies [88]. The same working group replaced shortenings in cookies with oleogels obtained by crude or refined soybean oil, structured by beeswax (10%, *w*/*w*), MAG (10% *w*/*w*), or their combination (each at 5%, *w*/*w*). Beeswax structured the oil in elongated needle-like crystals, while MAG crystalized the oil in spherulites shape. Novel cookies had comparable parameters with the commercial cookies (made with shortening) in terms of weight, thickness, width, spread ratio, and hardness [89]. Another interesting application of beeswax as an internal structuring compound and whey protein isolate (WPI) as an external coating was completed in fish oil for better oxidative stability by thermal treatment and ultraviolet C radiation [90]. 

Moreover, several working groups used combination of gelators, since one could not give the needed requirements. In that sense [91] prepared flaxseed oil oleogels with the aid of berry wax or sunflower wax with glycerol monostearate (GMS) at fixed total concentration of 6% (*w*/*w*). In this study they noticed that GMS addition decreased the oil binding capacity of oleogel structured with berry wax, and improved the properties in oleogels structured with sunflower wax. Combination of oleogelators was used in the study conducted by Choi et al. [92] at a fixed total concentration of 10% wt. The oleogel prepared by the candelilla wax (CDW) and glyceryl monostearate (GMS) at the proportion 3:1 demonstrated the eutectic behavior, harder texture and lower oiling off due to the formation of dense network with small crystals. A recent study showed that not all kind of oleogelators could structure oil with medium-chain TAG. In this study it has been shown that rice bran wax was not able to structure the liquid oil alone, whereas the addition of 5% (*w*/*w*) MG to sunflower wax and beeswax led to creation of self-standing gel. The strongest oleogel was obtained from beeswax and rice wax at concentration of 15% (*w*/*w*) [93]. Moreover, carnauba wax based oleogel was reinforced with adipic acid and was implemented in model cake and beef burger. The reinforcement (higher than 3%, *w*/*w*) produced oleogels with improved thermal, oxidative and crystallinity behavior as well as oil binding capacity caused by the formation of new intra- and intermolecular hydrogen bonds [94].

Ethylcellulose (EC) was used as a sole oleogelator in structuring soybean oil at a concentration 6% (*w*/*w*) [95] or in combination with candelilla wax and MAG [96]. According to the later study EC oleogels gave grainy texture, while the mixed oleogel showed better elasticity due to the formation of junction zones between EC and MAG.

Oleogels were prepared with emulsion templated approach when hydrophilic high molecular oleogelators (HMOG) were used. First, the oil-in-water emulsion was prepared using MAG as an emulsifier with the addition of vitamin C, followed by freeze-drying for water phase evaporation. The effect of different oil types (linseed oil, corn oil and camellia oil) and different crystallization temperature (−18, 0, 5 and 25 °C) was studied for oxidative stability in vitamin C loaded oleogel [97]. Emulsion based soybean oleogels were prepared with gelatin, Chinese bayberry leaves and different polysaccharides (pectin, xanthan gum and arabic gum) and successfully applied in cakes for margarine replacements [98]. Oils can be structured by whey protein isolate (WPI) produced as aerogels particles (by freeze-drying or supercritical drying). Better physical properties in terms of hardness and plasticity were exhibited by the oleogel structured with supercritical dried WPI due to the lower agglomeration of particles during preparation [99]. Guo et al. [100] studied the addition of trace amounts of water at different temperatures (20 °C, 45 °C, 70 °C and 95 °C) to the sunflower oil based oleogels gelled by a mixture of ceramide and lecithin. The addition of water at 20 and 95 °C led to formation of gels, where those at 95 °C were stronger but more fragile with the oil packed in droplets and stabilized by lamellar shells (lecithin) and fibrillar crystals (ceramide), while the samples prepared with the water heated at 45 °C, 70 °C were weak and unstable. In another recent study, aerogel from alginate/soy protein conjugates was prepared by freeze-drying with a good oil absorption and holding capacity [101]. One interesting research presented a development of bigel as a carrier for β-carotene. The bigel was obtained by mixing the hydrogel that was gelled with κ-carrageenan and the oleogel that was structured by MAG under temperature control. As presented by the authors, the mechanical properties (storage modulus, stiffness, fracture stress) and thermal stability of the bigels were improved when the fraction of oleogel was greater, whereas the highest crystallinity was observed in the bigel with 50% oleogel. A bigel with greater oleogel fraction could stimulate better release of β-carotene during simulated digestion [102].

### 3.2. Bioengineered Oleogels

Creating a sustainable development by using renewable resources is the future trend of oleogels production. The concept of Circular Economy by valorization of food industry side streams and by-products by means of biotechnology was extensively explored by Papadaki et al. [23,103]. This research group made successful attempts in creating novel oleogels using microbial oil produced from different industries side streams or by using gelators derived from fatty acid distillate [23,103,104,105]. The microbial oil produced by oleaginous yeasts and fungi in nitrogen-limited media is mostly used for biodiesel production. However, there are several reports in the literature where it is further enzymatically converted into wax esters by lipases in a solvent free system using cetyl, oleyl and behenyl alcohols [105].

According to Papadaki et al. [23] study, sugarcane molasses and soybean cake, by-products from food industry can be recycled into fermentation media and nutrient supplement, respectively for microbial oil production rich in carotenoids. The microbial oil represents the intermediate products that can be converted into wax ester via lipases in solvent free reaction and then employed as a structuring agent in oleogelation. Soybean fatty acid distillate (SFAD) derived from soybean oil extraction processes could be also enzymatically modified into wax ester. The microbial oil was produced in batch and fed-batch operation mode with the aid of the oleaginous yeast *Rhodosporidium toruloides* DSM 4444. Oleogels were produced with soybean oil and microbial oil structured either by SFAD cetyl wax ester or microbial oil cetyl wax ester. In the case of microbial oil batch production, it was observed that lipid production and cell concentration are affected by the addition of nitrogen source, trace elements and phosphate salts in the fermentation medium. Fermentation medium composition also affected fatty acid composition, thus by its optimization a microbial oil with appropriate composition can be produced. When fed-batch fermentation was performed with sugarcane molasses enriched with nitrogen source and trace elements, the highest microbial intracellular lipid concentration (54.6%, *w*/*w*) was noticed at 121 h with sugar to lipid conversion 0.13 g/g. On the other hand, when the fed-batch fermentation was performed on a soybean cake hydrolysate, the highest microbial intracellular lipid concentration (49.8%, *w*/*w*) was reached at 91.5 h with sugar to lipid conversion 0.15 g/g. In both fermentations at the point of the highest microbial accumulation, the highest carotenoid production was achieved. Both side streams proved good source of essential nutrients for efficient microbial oil production. In later stage microbial oil was converted to wax ester and used in oleogel production at high concentration of 20% which gave negative effect on the sensorial properties. Different waxes (microbial and SFAD wax) contributed to different crystal morphology. More precisely, SFAD wax gave larger flake-liked shape crystals and microbial wax provided spherulite-shaped crystals which tended to agglomerate. It was concluded that the different crystal formation comes from waxes’ different degree of saturation. Oleogels had similar melting temperature, lower that the human body, suggesting its potential use in food products. Soybean oil-microbial wax based oleogel had high stable and compact crystalline network indicated by high G’ values due to the presence of mixture of C16 and C18. At a certain temperatures oleogels had solid-like properties expressed by G’ > G”, whereas at elevated temperatures a transformation of the gel into sol was observed expressed by G” > G’. Microbial oil-microbial oil wax based oleogel exhibited the highest firmness, while soybean oil-SFAD wax oleogel decreased 82% in firmness until the 20th day. Thus, by tailoring oleogelators with aid of biotechnology, oleogels with different thermal, rheological and textural properties can be produced, opening their application possibility in different food. Similar study was published by the same research group where the obtained wax esters from microbial oil were used as structuring material in virgin olive oil based oleogel [104]. The microbial oil was fermented in batch operational mode by oleaginous yeast *Rhodosporidium toruloides* DSMZ 4444 grown on cane sugar medium supplemented with nitrogen source and trace elements. The hydrolysis of the microbial oil was catalyzed by vegetable enzyme from castor bean seeds and the esterification between hydrolysates and fatty alcohols (oleyl or cetyl) was performed by lipase B from *Candida antarctica*. Olive oil based oleogels were prepared by the aid of cetyl-derived wax ester in different concentrations (7%, 10% and 20% *w*/*w*) by the dispersion technique under agitation at high temperature, followed by cooling. The highest intracellular content of microbial oil (34%, *w*/*w*) occurred at 89 h after the depletion of nitrogen source and was composed of oleic, palmitic and stearic acid. The microbial oil hydrolysates, oleyl, and cetyl alcohols were the substrate for the production of wax esters. The best yield in the esterification process was obtained at 40 °C for the oleic esters and at 50 °C for cetyl esters in a solvent free process. After the addition of limonene (30%), the highest conversion yields for both esters were noticed at 50 °C. In the same study, the potential application of produced wax esters as oleogelators was examined. Only the concentration of 20% cetyl wax esters out from all tested was sufficient in structuring the oil in solid-like state. The oleogels had large flaked-like crystals up to 100 μm, due to the cooling method employed in the gel preparation or the oil fatty acid composition and crystal polymorphism. The melting temperature of the oleogel was affected by the melting temperature of the gelator and was lower than the human mouth, which showed potential application in confectionary products. By increasing the temperature, the viscosity of oleogels decreased, transforming itself from solid-like state into liquid-like state. At room temperature, G′ was greater than G″ indicating solid elastic behavior of the oleogel. However, the crossover point (G′ = G″) was observed at 33.7 °C where crystalline fats were melted. The novel oleogel had potential application in margarine and spreadable products. In that sense, the texture of oleogel was compared to breakfast margarine. It had good and comparable firmness when freshly produced but decreased by 60% after 20th day of storage attributed to the large crystals and weak internal strength. In a recent study Papadaki et al. [103] evaluated the ability of enzymatically synthesized SFAD wax esters to structure extra virgin olive oil. Their motive was again the utilization of industrial side stream derived from the soybean oil refining process in the production of novel olive oleogels. Wax esters were enzymatically obtained from SFAD and cetyl alcohol in the molar ratio 1:1 in the solvent-free reaction, while the olive oil based oleogel was prepared by employing different concentrations of the enzymatically obtained wax ester by the dispersion method at high temperature. The concentration of 20% *w*/*w* was taken as optimum resulting in structured lipids. The crystal morphology of the obtained oleogel was comparable to the oleogels formulated by soybean oil and SFAD wax esters [23], which confirms that the crystallinity is highly affected by the type of the oleogelator. The oleogel had high intensity of the yellow color that was close to commercial spreadable products. The melting temperature was observed at 29.8 °C, which clearly indicates its potential use in food products. Table 2 summarizes various oleogels as fat replacers in food products published in the last three years. The oleogel showed solid-like properties at the temperature range of 20–27 °C and a crossover point at 28–29 °C where it transforms from gel into sol, which was a bit lower compared to their previously formulated oleogels by soybean oil and SFAD wax esters. The firmness was again the highest for freshly produced oleogel, which subsequently decreased over time, which again is attributed to large crystals and less contact points among them. They proposed application of current oleogel in mixture of other oleogels, hard fats or high and low melting waxes, or as carriers of bioactive compounds.

#### Developing Emulsions Using Enzymatically Modified Emulsifier 

Emulsion represents a system containing two immiscible liquids one being dispersed and the other continuous phase. According to that, emulsions can be divided in three categories: water-in-oil emulsions (dispersed phase is water), oil-in-water emulsions (dispersed phase is oil) and complexed emulsions, such as water-in-oil-in-water emulsions. For a formation of stable emulsion, a shear force and a presence of surface-active compound are needed. The droplet size depends on the amount of shear i.e., the higher the shear the smaller the droplet. In addition, the compactness of the emulsion depends on the type and quantity of the emulsifier. The emulsifiers are divided in two groups: fine solids (materials wetted by both water and oil) and surface-active agents (materials soluble in both water and oil). For the food industry, surface-active agents are of a greater importance. They are composed of hydrophilic component (affinity towards water) and hydrophobic component (affinity towards oil). Their mechanism in creating a stable emulsion is by reducing the interfacial tension at the oil-water interface [107]. 

Over the past years a lot of research was focused in developing biodegradable emulsifiers such as MAG and TAG. They are generally produced by esterification and glycerolysis, but added to an emulsion system separately. In an interesting research, Kowalska at al. [106] demonstrated the possibility of producing TAG, DAG and MAG in sufficient amount at the same time to stabilize an emulsion system, where MAG and DAG play a role of emulsifiers, using mutton tallow and hemp oil. The process was achieved by creating imbalance between the interesterification and the hydrolysis of fat using additional amount of water. In their study, they optimized the amount of water that can efficiently activate the lipase. The biocatalyst extracted from *R. miehei* was sn-1,3 regiospecific. The fatty acid composition of raw non-interesterified lipids showed no presence of MAG or DAG, while the fatty acid composition changed in interesterified fat in favor to polar compounds. The addition of water in the reaction mixture increased the content of DAG, MAG and FFA indicating that the ratio of polar to nonpolar fractions strongly depends on the water content in the system. Interesterified fats showed decrease in slip melting point compared to the mixture before esterification, due to the redistribution of fatty acids. These changes led to changes in kinetics of crystallization, structure and rheology properties in interesterified fats, properties that highly influence sensory attributes and overall acceptability of the fat products. Noninteresterified fat was composed of asymmetric spherulites closely bound with crystals in a shape of long needles. Esterified fats consist symmetrical, regular round shapes densely packed. These different samples of interesterified/non-interesterified fat were used as emulsifiers in water-in-oil emulsion stabilized by carboxymethylcellulose (CMC). Emulsion texture was analyzed on freshly prepared emulsions and on stored over 30 days. Freshly prepared emulsions that contained interesterified fat were softer, than those that contained noninteresterified blend. In contrary, stored emulsions had firmer texture, especially the formulations that contained the highest content of MAG and DAG. The formulation that stood out in firmness was the sample prepared with lecithin. Freshly prepared emulsions containing interesterified fats were less spreadable than the commercial products. Spreadability of the prepared emulsions decreased over time, while the adhesiveness increased. The pH determined for all emulsion formulations was in the range of 6.2–6.9. The samples were analyzed for the destabilization phenomena or separation that can occur in the system using Turbiscan Lab. These phenomena include creaming, sedimentation, coagulation, flocculation or Ostwald ripening. It was observed that almost in all samples drops of the dispersed phase are merging which results in coalescence progressing to a separation of emulsion phases. Formulations that contained insufficient amount of emulsifiers (close to the limit concentration that would allow stabilization) broke down over time, while visually no significant instabilities were observed. The most stable formulations were those prepared with the highest content of DAG in situ. The droplet size that was found to be in a range 3.4–14.8 mm remained unchanged during the storage period for the emulsions with EIE fats in highest content of produced emulsifiers in situ. The viscosity of the EIE samples ranged between 1014 and 2033 cP, while for NIE sample emulsified with lecithin, the viscosity was 5803 cP. It was concluded that the most stable emulsion was the one obtained with a synergistic effect of the emulsifier and viscosity modifier (CMC). The rheological behavior of the emulsion systems depends on the amount of DAG and MAG created during the interesterification process. The formulations containing higher fractions of enzymatically obtained emulsifiers had higher G’ modulus in comparison to the ones with lower fraction, which shows formation of good microstructure. When the emulsions were compared according to their viscoelastic behavior using a damping factor (gives information about the strength of the interactions) it was estimated that the reference formulation stabilized with lecithin was the most rigid one, while formulations containing EIE fats show lower rigidity. By adequately engineering the process of simulant transesterification and glycerolysis it is possible to obtain stable emulsion as an alternative for the cosmetic industry. 

Emulsions (type oil-in water) were made from enzymatically interesterified blend of mutton tallow (MT) and hemp seed oil (HSO) in different proportions stabilized with xanthan gum and scleroglucan [18]. Before creating the emulsion systems NIE and EIE blends were characterized in terms of acid value, melting point and the quantity of MAG and DAG. As expected, the acid value of the EIE fats sharply increased indicating the higher content of the polar fraction in the blend. If in the reaction mixture proper amount of water is added, the reaction can be shifted from interesterification towards hydrolysis. The obtained EIE blend has a built-in emulsifier that can be used for ready-made emulsions. The melting point of EIE blend decreased compared to the NIE fat, especially for the fat in proportion 3:1 MT: HSO. Formulated emulsions were characterized in terms of color, where a significant change can indicate a destabilization of the system. The researchers observed slight change in L*, a* and b* parameters after the storage period. After this period the emulsions were lighter in color (increased L* values), slightly greener (decreased a* values) and with a slight blue shade (decreased b* values). For the freshly prepared emulsions, the b* values increased with increasing the concentration of the texture modifier and the fraction of the liquid oil. The Chroma (C*) also changed by using the higher content of the polysaccharides and by aging of the systems. The formulations containing highest fraction of liquid oil exhibited the smallest changes in the color intensity. All of these changes detected by instruments were not visually noticeable. The emulsions revealed pseudoplastic behavior which can break down (easy skin application) when a shear is applied. The formulations with increasing texture modifier had higher apparent viscosity. Modification of fats changed the SFC and the crystalline structure, thus the rheological properties of the blend. A high viscosity and stable product were also observed in the formulations with higher fractions of mutton tallow. The highest viscosity exhibited the system containing mutton tallow and hemp seed oil in proportions 3:1 *w*/*w* and a stabilizer in a concentration of 1% *w*/*w*. The prepared emulsions showed a homogenous distribution of the lipid droplets without agglomerations and irregularities. The average droplet size of the produced emulsions was between 2.2 and 13.9 μm with the smallest droplets obtained when higher amount of texture modifier and higher proportion of hemp seed oil were used [18]. This report presents another successful incorporation of biotechnology in structuring lipids with a specific purpose to be used in a cosmetic, pharmaceutical or food product.

## 4. Fat Replacers of Nonlipid Origin

In previous sections, the fat substitutes based on lipid origin were discussed. They can fully replace the conventional fat, since they possess similar physical and functional characteristics due to their nonpolar compounds. However, fat mimetics based on nonlipid origin are generally polar compounds that can imitate some of the textural and organoleptic characteristics of conventional fat, hence they are used for partial replacement of fat in food product. One of their most pronounced characteristics is the water-binding capacity, which mimics the creaminess attribute found in full-fat products. Yet, mimetics main disadvantage is the lack of flavor carrying capability. Fat mimetics consist of two large groups: protein-based and carbohydrate-based. For protein-based mimetics, proteins are transformed into gel network by microparticulation, which contributes to the smooth, creamy texture in the mouth. These types of mimetics are not suitable for harsh cooking regimes like frying due to the protein denaturation. Generally, they are derived from egg, milk, pea, wheat or soy proteins and are applied in low-fat cheese, mayonnaise, salad dressings, ice creams, sauces, dips and baked goods [108,109,110,111,112,113,114]. On the other hand, carbohydrate-based mimetics also create gel-like structure by incorporating large amount of water, which provides increased viscosity, thickness and creaminess similar to the full-fat product. Apart from their non-appropriateness to be used frying applications, they also reduce the flavor intensity. 

Most common carbohydrates used as fat mimetics include gums, cellulose, starches, dextrins and maltodextrins, polydextrose, inulin etc. [26,28,30,115,116,117]. 

Although the trend of fat replacements in different food products formulations among researchers started decades ago, the challenge of creating reduced fat original-like product without sacrificing the taste and functionality still persists. Various research groups are employing novel strategies in creating the best composition that will mimic conventional fat without repulsing consumers. In this review, we are going to discuss the very recent achievements and approaches in fat mimetics from nonlipid source. 

Microparticulated whey proteins (MWP) are produced from ultra-filtered whey proteins using high temperature and high homogenization pressure or by liquid extrusion. The processing parameters for MWP fabrication were optimized by factorial design and their application in spread and petit-Suisse cheeses was evaluated [118]. It was concluded that fat partial substitution (until 40%) with MWP did not alter the textural characteristics compared to the control, while on the basis of the sensory test, newly developed products were highly acceptable. Liquid extruded MWP showed viscosity particle size dependence, reduced rate of syneresis, increased creaminess and higher firmness when applied in yogurt and compared to market fat-reduced yogurts [119]. By changing the mode of processing, the microparticulate size can be adjusted to produce certain viscosity and creaminess regarding the product application. One recent study shows the possibility of 3-D printing of whey protein gels where different parameters were examined including ionic strength, protein content, fat content, and partial substitution of whey protein with MWP. Gels were characterized in terms of texture and rheology, where it was confirmed that all the modifications were not sufficient for continuous extrusion process. Fat addition increased stiffness of the gel, however for better printability softer and less-elastic gels were needed. That was achieved by substituting whey proteins by MWP which resulted in stable shapes after deposition [120]. A bioeconomical approach of valorizing side stream from dairy industry was implemented in production of reduced fat washed curd cheese. The by-products, previously concentrated by ultrafiltration comprising concentrated whey, buttermilk and sheep second cheese whey were employed in reduced fat cheese at the level of 5%. The protein-to-fat ratio was found lower in full fat control sample than in the different reduced fat cheeses. The most similar novel product to full fat product was the buttermilk reduced fat cheese in terms of hardness, and chewiness which was also sensory acceptable evaluated by the panelists [110]. Studies have shown that the phosphorylation modification technology of plant proteins such as pea protein isolate, peanut protein isolate, and soy protein isolate could ameliorate their functional properties (solubility, emulsifying property, emulsifying stability, foaming property and oil absorption capacity). Liu et al. [111] presented that modified pea protein isolate forms smooth uniform lamellar structure compared to the irregular block structure of unmodified pea protein isolate. Together with xanthan gum (0.4%, *w*/*v*), pea protein isolate can produce effective fat mimetic that can substitute up to 20% of the light cream in mango mousse cake. In order to moderate the rigid and rubbery texture that appears in low fat cheese, Paximada et al. [112] employed double plant-based protein emulsions embedded in the cheese protein matrix that will act as an active filler. Double emulsions were prepared from whey protein isolate, pumpkin seed protein or rice protein dissolved in water as the inner aqueous phase, anhydrous milk with PGPR as the oil phase and milk as the outer aqueous phase. The type of protein and its concentration affected the viscosity and the droplet size in emulsions. Protein based fat replacers, classified as animal and plant proteins were exhaustively discussed by Yashini et al. [121]. They presented nice comparative study between the two groups of protein fat replacers related to the extraction methods, functional properties and their effects in low-fat food products. Furthermore, Paglarini et al. [109] reviewed protein-based hydrogel emulsions, their methods of production and nutritional benefits as fat replacers in meat products.

Carbohydrates are being used to replace fat in products for decades due to their ability to create fat-like sensation and their less energy contribution than fat. Among them starch-based replacers are the most common either in their natural form or chemically/enzymatically modified. Recently, modified arrowroot starch was employed as a fat replacer in mayonnaise at levels of 30% and 50%. Physicochemical and pasting properties were dependent on the modification technique (octenyl succinic anhydride, annealing, citric acid hydrolysis, acetylation, and heat moisture treatment). Low-fat mayonnaise had better emulsion stability and higher viscoelastic properties [26]. In general, starch granules can mimic the fat globule giving the appropriate structure or create thermoreversible gel contributing to the fat-like mouthfeel. A short review the starch-based mimetics was given by Chen et al. [115]. Alternative carbohydrate that can take the role of a fat replacer is inulin, which can give additional functionality to the product due to its prebiotic characteristics. When mixed with water, it creates gel network that produces fat-like mouthfeel sensation [30]. It is mostly used in gelled emulsion where PUFA is embedded. In fermented sausage, 64% pork back fat was successfully replaced by inulin in a form of dispersion or linseed oil gelled emulsion. Texture differences were more pronounced in the emulsion form fat replacer [28]. As an emulsion with soybean oil, inulin was implemented in bologna sausages, where the novel product had higher quantity of PUFA, lower SFC and was more stable to heat treatment, however the textural properties were negatively affected [116]. Inulin used in combination with hydroxypropyl methylcellulose at appropriate concentrations applied in low-fat muffins can reduce the viscosity of the batter and improve the textural characteristics by reducing the degree of retrogradation and the hardness of the product [30]. Shortbread cookies were produced in the same fashion of reduced fats by substituting them with inulin-extra virgin oil emulsion gel at different levels (0%, 20%, 40% and 50%). As the fats were reduced, the texture of the cookies hardened, the volume increased, and the color become darker [122]. In another study, the cake recipe was reformulated by simultaneously reducing the sucrose with Rebaudioside A and fat with inulin. The viscosity of the batter was determined by water as a prime component, followed by sucrose and inulin, whereas oil played a big role in the consistency [123]. To compensate for the loss of the structure due to the fat reduction in cocoa fillings, cellulose ether-based hydrogel emulsions with viscoelastic properties are being used. Their different chemical substitutions influence the temperatures of gelation and shear thinning characteristics at room temperature [117]. An overview of the different fat mimetics, their applications and processing parameters is given in Table 3.

An interesting research was conducted by partially or fully substituting the fats in beef burgers with oat-hull based ingredients while enriching them with beta-glucans to a content near the recommended daily intake. The burger showed an oil-retaining effect, softer texture and better juiciness [125]. 

Moreover, Schadle et al. [113] used numerous carbohydrate and protein-based fat mimetics in a combination with rennet casein to study their effects on the physical and textural behaviors of low-fat cheese. The flowability can be decreased by using inulin, and corn dextrin, a microparticulated whey protein, increased the storage and loss moduli, whereas the increased concentration of rennet casein made the product harder. Thus, by using appropriate combinations and concentrations of different fat replacers, an engineered low-fat cheese with desirable sensory characteristics can be produced. Different carbohydrate-based fat mimetics, their effects on food quality and certain application were reviewed by Peng at al. [126].

## 5. Conclusions

Lipid structuring as an idea was developed decades ago, leading to the extensive exploration and industrialization of the techniques, which nowadays are being used in the production of margarines, shortenings and creams. These conventional techniques produce large amounts of *trans* fats linked to negative effects in human well-being. Therefore, the interest in finding alternative ways of tailoring lipids that will have the functional properties of saturated fats but will have the nutritional profile of mono- and polyunsaturated fats have risen both in academia and the industry. For the comprehensive conclusions on all the discussed techniques, Table 4 was organized in a very systematic manner while critically assessing the advantages, limitations, impacts on human nutrition and the industrial applications of the conventional and unconventional techniques for tailoring the structures of lipids, oleogels and fat replacers.

To resolve the issue of *trans* fats is a big challenge. As reviewed in this paper, a number of various techniques are being explored, including enzymatic esterification, genetic modification of the lipid in situ, oleogelation, using fat replacers from lipid or nonlipid origin etc. Although, for each of these techniques, promising results have been reported, they all have their own limitations, with scaling-up being the common problem for all of them. The enzymatic interesterification is dealing with poor enzyme stability, the process is costly and there is a lack of research on how the positional composition of TAGs affect the digestibility and lipid metabolism. The main concern of genetically modified lipids, besides the high cost of raw materials, the time-consuming process and the possibility of crosspollination of the neighboring crops, is the regulatory aspects of genetic modifications and the hesitant attitudes of consumers. Oleogelation itself developed various approaches based on the processing methods (direct dispersion, oil binding method, emulsion template method and biphasic systems) or on the structurant origins (low molecular weight/high molecular weight). Oleogels are being successfully applied in water-free and water-containing products. However, research is still needed in exploring the best suitable oleogelator that will be food-grade, economical and efficient at low concentrations. One type of gelator of this kind is natural wax; however, the produced oleogels have low stability during storage due to crystal aggregation. Polymer oleogels have a better stability but require a lot of processing steps, while the combination of both leads to the formation of weak gels. On the other hand, biphasic systems are very specific to the product type, but they rarely match the expected sensation by the consumer. The compatibility between oleogels and the food matrix still needs to be studied. Protein- and carbohydrate-based fat replacers can mimic conventional fat to some extent and are better applied in multicomponent systems, while their incorporation in high-fat systems is difficult to achieve. Moreover, these types of fat replacers are not suitable for specific technological operations or harsh cooking regimes. For all the structuring methods, extra research is needed to explain the relationship between the process, the function of the tailored lipid and the structure of the food matrix. Besides this, the future trend in oil structuring might be the use of sustainable oils (i.e., microbial oils) and enzymatically obtained wax esters via the valorization of food industry byproducts. 

## Figures and Tables

**Table 1:** Enzymatic interesterification of different lipids and their applications, health and functional benefits.

Enzyme	Sources of Lipids	Application	Health and Functional Benefits	Ref.
Lipozyme TL IM lipase	anhydrous milk fat and palm olein	probiotic table spread containing milk fat	increased unsaturated fatty acids, good probiotic viability, improved melting behaviour	[60]
Lipase form porcine pancreas	beef tallow and rice bran oil	new edible fat formulation	*trans* free fats, decreased saturated fats	[62]
Lipozyme TL IM (*Thermomyces lanuginose*)	hybrid palm stearin and palm kernel oil	new edible fat formulation	higher content of unsaturated fatty acids, decreased melting and crystallization temperatures	[61]
Lipozyme TL IM (Thermomyces *lanuginosus*)	high oleic sunflower oil (HOSO), and fully hydrogenated soybean oil (FHSO)	fats for confectionery and bakery products	lower acyl migration of sn-2 position of the unsaturated fatty acid, and better nutritional value	[57]
1,3-specific lipase (*Rhizomucor miehei*)	milk fat and rapeseed oil	model meat batter from chicken breast	higher content of unsaturated fatty acids, lower induction times, lower apparent viscosity	[63]
Lipozyme TL IM (*Thermomyces lanuginose*)	palm olein, fully hydrogenated palm oil and palm kernel oil	cocoa butter substitutes	no tempering is required	[64]
Lipozyme RM IM *(R. miehei)*	*Cinnamomum camphora* seed oil and fully hydrogenated palm oil	cocoa butter substitutes	low-cost and low-calorie chocolate	[14]
Lipozyme TLIM *(Thermomyces lanuginose)*	palm stearin fraction and fish oil	substitute milk fat in infant formula	rich of n-3 PUFAs	[12]
Lipozyme RM IM, Lipozyme TL IM, NS 40086	Catfish oil and coconut oil	substitute milk fat in infant formula	obtained similar TAG composition as human milk	[65]
Lipozyme 435	soybean oil and fully hydrogenated palm oil	margarine analogues to beef tallow	low *trans*-fatty acids with desired spreadability	[11]
Novozym 435, Lipase PS “Amano” IM Lipomod TM 34P	palm stearin and canola oil	frying shortenings	zero-*trans* structured lipids used for high temperature applications	[66]
Lipozyme TL IM	palm stearin and soybean oil palm stearin and rapeseed oil	interesterified blend-based fast-frozen special fat	better storage quality for palm stearin and rapeseed oil blend	[67]
*C. antarctica* lipase B	cottonseed oil	margarine and peanut butter	higher crystallization temperature, homogenous crystal network, reducing LDL and total cholesterol	[16]
*C. antarctica* lipase	palm stearin-olein blend	emulsifier	higher melting point and hardness than the liquid oil, stable emulsions	[68]

**Table 2:** Various oleogels as fat replacers in food products—an overview of the last 3 years of published papers.

Fats	Gelator/ Concentration	Application	Structuring Methodology	Ref.
rice bran oil	rice bran wax 0.5–25% (*w*/*w*)	new edible fat formulation	dispersion of wax into hot oil followed by cooling	[83]
flaxseed oil	berry wax or sunflower wax with glycerol monostearate (GMS) 6% (*w*/*w*)	new edible fat formulation	dispersion of wax and GMS into oil followed by cooling	[91]
grapeseed oil	candelilla wax and glyceryl monostearate (GMS) 10% (*w*/*w*)	new edible fat formulation	dispersion of gelators at 90 °C, followed by cooling	[92]
canola oil	candelilla wax 5% (*w*/*w*)	sponge cakes	dispersion of gelator at 160 °C, followed by cooling	[84]
linseed oil with a high content of linolenic acid	beeswax 8% (*w*/*w*)	meat-based spreadable product	dispersion of beeswax under stirring at 80 °C, followed by cooling	[85]
crude or refined soybean oil	beeswax BW monoglycerides MAG 10% (*w*/*w*)	cookies	dispersion of the oleogelators above the melting temperature at 140 °C, followed by cooling	[89]
corn germ oil	rice bran wax (RBX) 3, 5, 7, 9% (*w*/*w*)	cookies	dispersion of rice bran wax at different concentration at 120 °C under constant stirring, followed by cooling	[88]
conventional sunflower oil or high oleic sunflower oil	glyceryl monostearate (GMS) 5% (*w*/*w*)	Bologna sausages	heating the mixture of oil phase (SF or HOS) and GMS at 90 °C, followed by cooling	[86]
linseed oil	beeswax and a mixture of γ-oryzanol and β-sitosterol 60:40 (*w*/*w*) 8% (*w*/*w*)	dry-cured sausages	heated with stirring at 80 °C until solubilization, followed by cooling	[87]
medium-chain triacylglycerides	rapeseed wax RAW, rice wax RW, sunflower wax SW, beeswax BW, monoglycerides MG, and c-oryzanol and b-sitosterol mixture c+b 5–15% (*w*/*w*)	new edible fat formulation	heated to the melting temperature according to the gelator, followed by cooling	[93]
soybean oil	ethylcellulose (EC) 0–10% (*w*/*w*)	new edible fat formulation	heated to the glass transition temperature of EC, followed by cooling	[95]
high oleic safflower oil	ethylcellulose monoglycerides candelilla wax	edible shortening	dissolving oleogelators in ethanol, evaporating the solvent, dispersing, and dissolving the solid residue in the vegetable oil	[96]
fish oil	beeswax for internal structure 5% (*w*/*w*) whey protein isolate (WPI) for external coating 2% (*w*/*w*)	new edible fat formulation	2% phospholipid was dissolved and mixed with fish oil under high shear; the structured oil was mixed with WPI solution; the external coating was formed by electrostatic deposition	[90]
soybean oil	carnauba wax (CW)- with adipic acid (AA) total at 6% (*w*/*w*)	cake and beef burger	mixture heated until complete dissolution at 150 °C, followed by cooling	[94]
mixture of corn oil, camellia oil or linseed oil	monoglyceride stearate + Vitamin C	new edible fat formulation	the mixture was heated at 80 °C and stirred, freeze-dried and crystallized at 0 °C	[97]
soybean oil	gelatin (2.5%, *w*/*w*) proanthocyanidins from Chinese bayberry leaves (BLPs) 4.0% (*w*/*w*), xanthan gum, pectin and arabic gum 10% (*w*/*w*)	cakes	mixture of gelators was stirred at room temperature until generation of colloidal complex than used in soybean oil for the preparation of O/W emulsion under high pressure, solutions of polysaccharides added to the emulsion and lyophilized	[98]
sunflower oil	whey protein isolate (WPI) hydrogels	new edible fat formulation	aerogel particles obtained by freeze drying or supercritical drying of whey protein isolate (WPI) hydrogels dispersed in oil	[99]
sunflower oil 85%	ceramide (CER) lecithin (LEC) total 15% (*w*/*w*)	new edible fat formulation	solubilization of oleogelators in oil, followed by cooling; water addition at different T to the sample followed by homogenization	[100]
corn oil	alginate/soy protein conjugates	new edible fat formulation	fabrication of alginate/soy protein conjugates aerogel by freeze-drying, followed by immersion in corn oil	[101]
corn oil	GMS 20% (*w*/*w*) β-carotene powder 0.1% (*w*/*w*) κ-carrageenan 1.5% (*w*/*w*)	bigel	oil phase: heating the oil at 80 °C for oleogelator dissolution, then at 140 °C for β-carotene dissolution, followed by cooling water phase: dispersion of κ-carrageenan at 80 °C, followed by cooling mixing both phases at 80 °C by stirring, followed by cooling	[102]
soybean oil microbial oil	SFAD cetyl wax ester or microbial oil cetyl wax ester 7, 10, 20 (*w*/*w*)	new edible fat formulation	mixture heated at 90 °C under agitation, followed by cooling	[23]
virgin olive oil	microbial cetyl-derived wax ester 7, 10, 20 (*w*/*w*)	new edible fat formulation	mixture heated at high temperature under agitation, followed by cooling	[104]
olive oil	SFAD cetyl wax esters 7, 10, 20 (*w*/*w*)	new edible fat formulation	mixture heated at 90 °C under agitation, followed by cooling	[103]
mutton tallow and hemp seed oil	TAG, DAG and MAG	in situ emulsion	high stable emulsion with good homogeneous dispersion of the droplets	[106]

**Table 3:** An overview of the different fat mimetics, their applications and processing parameters.

Fat Mimetic Type	Application	Processing Parameters	Ref.
microparticulated whey proteins (MWP) 25 and 100% MWP (spread cheese) 25, 50 and 100% MWP (Petit-Suisse cheese)	spread and petit-Suisse cheeses	microparticulation: 90 °C and 140 bar	[118]
liquid extruded MWP	stirred yogurt	hot extrusion process and protein concentration using a co-rotating twin-screw extruder	[119]
microparticulated whey proteins (MWP)	/	concentrating the whey protein cluster by ultrafiltration and thermomechanical processing of the resulting concentrates	[124]
microparticulated whey proteins (MWP)	3-D printed gel	printing at room temperature using an extrusion nozzle of 1.6 mm in diameter, printed layer by layer, total of 10 layers 8% WPI/2% MWP and 6.5% WPI/3.5% MWP	[120]
concentrated whey, buttermilk and sheep second cheese whey; concentration of 5%	reduced fat cheese	ultrafiltration process temperature (40–45 °C) membrane cut-off (10 kDa) CW—thermal denaturation of retentate (90 °C for 20 min), homogenization at 10 MPa	[110]
phosphorylated pea protein isolate	mango mousse cake	phosphorylation modification: pH 12, temperature at 70 °C addition of 7.0% (*w*/*v*) sodium tripolyphosphate (STP)	[111]
whey protein isolate (WPI) rice protein (RP) pumpkin seed protein (PSP) 10, 15, 20, and 25%, *w*/*w*	low-fat cheese	primary emulsion: preparation of WPI, RP and PSP stock solutions (hydration), preparation of fat phase with lipophilic surfactants (2%) in the ratio 1:1 40% of aqueous phase added to the fat phase, homogenized at 45 °C for 20 min at 25,000 rpm, followed by cooling double emulsion: dispersion 5% of primary emulsion in 95% external aqueous phase, followed by homogenization at 8000 rpm for 10 min	[112]
dietary fibres from black bean coats and gelatin	meat balls	gel preparation: dietary fibers from black bean coats and gelatin heated at 55 °C for 15 min, followed by cooling, cross-linked by calcium chloride, transglutaminase or combination of both	[25]
inulin, corn dextrin, polydextrose, and MWP combined with rennet casein	low-fat cheese	pre-hydration of fat replacers and their dispersion in a blend of skim milk powder, trisodium citrate, sodium chloride, citric acid, lactose before the addition to the minced cheese	[113]
modified arrowroot starch	low-fat mayonnaise	octenyl succinic anhydride, annealing, citric acid hydrolysis, acetylation, and heat moisture treatment	[26]
inulin	fermented sausage	inulin gelled suspension dissolved in water with pork gelatin, heated and mixed inulin oil gelled emulsion prepared from previously made pre-emulsion (water, oil and emulsifier) added to inulin gelled suspension and mixed at ambient temperature	[28]
inulin	Bologna sausages	emulsion gel preparation: 4% soy protein 50% soybean oil, 16.5% inulin and 29.5% water mixed	[116]
inulin	shortbread cookies	extra virgin olive oil, inulin, soy lecithin and water were mixed and homogenized for 5 min with high power ultrasound using a 200 W transducer	[122]
inulin hydroxypropyl methylcellulose 0.2, 8%	muffins	inulin and hydroxypropyl methylcellulose at appropriate concentration were added to the muffin batter recipe	[30]
inulin Rebaudioside A	cake batter	inulin and Rebaudioside A at different levels were mixed with other components of the cake batter	[123]
methylcellulose, MC, hydroxypropyl methylcellulose, HPMC,	cocoa filling	preparation of emulsion: dispersing of cellulose ether in the sunflower oil with the stirrer at lowest speed, followed by adding water gradually to the previous dispersion while stirring, obtaining the hydration of the cellulose ether and homogenizing for 15 s at 6500 rpm and 60 s at 17,500 rpm	[117]
oat-hull-based ingredient	beef burger	dispersing the ingredient in water, followed by mixing for 5 min at 13,500 rpm after which the gel was cut into small pieces to be used, freshly prepared	[125]

**Table 4:** Critical assessment of the conventional and unconventional techniques for tailoring the structures of lipids, oleogels and fat replacers.

Technique	Advantage	Limitation	**Impact on Human Nutrition**	**Commercial Products**
CONVENTIONAL				
**Fractionation**	prevent (olein fraction) or ensure (stearin fraction) solidification at ambient T enrichment of the oil with unsaturated/saturated TAGs obtaining of fractions with narrow melting point	sustainability issues linked to tropical fats high use caused by the high demand of solid fat big capital costs high saturated fatty acids (SFA) of stearin fraction	no changes in TAG composition, thus no adverse effects on humans health on balanced diets high intake rates of foods containing stearin fraction are linked to coronary diseases	frying oils salad oils salad dressing salad mayonnaise formulation cream ice cream shortenings buttercreams infant formulas chocolate
dry	minimum operating costs high olein yields	incomplete phase separation—“entrapment” of oil		cooking and salad oils
solvent	less viscous suspension faster crystallization smaller crystallizer volume high separation efficiencies	recovery of the solvent flavor problems associated with solvent residues		cocoa butter replacers
detergent	transfer the crystallized material from the oil phase to the aqueous phase in order to facilitate subsequent separation			milk fat fractionation
**Hydrogenation**	increases the melting point of the fat change liquid oil into solid fat stops the decomposition or rancidity of unsaturated fats	catalyst selectivity catalyst deactivation during storage mass transfer limitations	formation of *trans*-fatty acids correlated to coronary diseases increase low-density lipoproteins (LDL), and decrease good high-density lipoproteins (HDL) side effects of *trans*-fatty acids: allergic reactions, arteriosclerosis, risk of cancer, decrease in insulin response, and slight immune dysfunction	dressings for salads cooking and frying products bakery coatings emulsions and spreads chocolate and confectionery products coffee creamers fried foods ready-to-use dough packaged snacks
hydrogenation with sc CO_2_	enhance the mass transfer enhance hydrogenation efficiency decreasing oil viscosity milder conditions of processing	pure CO_2_—low solubility for higher fatty acids high pressure is required to achieve supercritical conditions, which significantly increase the cost	reduced *trans*-fatty acids content in hydrogenated oils and fats; however, health concerns still present	
**Chemical Interesterification** **(CIE)**	modification of the physicochemical properties of fatty materials change the overall melting profiles to improve the compatibility of the different TAGs in the solid state modification of the plasticity of a product by changing its crystallization behavior	drastic operational conditions high temperature or pressure degradation of fats and introduction of side products	possibility of acute health effects due to the random esterification	spreads, bakery products confectionary products infant formula (Betapol)
**NONCONVENTIONAL**				
**Enzymatic Interesterification** **(EIE)**	high selectivity mild reaction conditions less side reactions less waste and ease of product recovery environmental benefits such as elimination of the use of potentially toxic chemicals and elimination of by-products and waste	high cost of the enzymatic process poor enzyme stability poor oxidative stability of the structured lipids	improved absorption/digestibility of saturated fatty acids possibility of negative affect on lipoprotein metabolism, glycemic control, immune function, and serum liver enzymes due to the alterations of FA on glycerol backbone	margarine shortening research studies: plastic fats human milk fat substitutes low calorie structured lipids cocoa butter substitutes and cocoa butter equivalents edible film applications
**Genetic modification**	compositional modifications of crop plant oils through traditional breeding techniques higher proportion of lauric acid eliminates the need of hydrogenation producing oil with higher fraction of SFC and higher melting point	possibility to compromise the ability of the seed to grow and develop limitation the crop yield induction of cross-pollination affect biodiversity the cost of raw materials regulatory issues the acceptance of the consumers	better oxidative stability in deep frying applications extended shelf life zero *trans*-fat low saturated FAs high oleic content liquid at room temperature excellent taste and flavor	high- oleic sunflower oil high-lauric canola oil (Laurical^®^) high-oleate phenotype of cultivated peanut research studies: human milk from model oilseed *Arabidopsis thaliana*
**Oleogelation**	transformation of liquid oils into a gel like structure which has rheological properties, viscoelasticity, spreadability, and firmness of a solid fat without containing large amount of saturated fats formation of three-dimensional gel network which mimic TAG crystallization prevention of oil migration between lipid-containing phases of composite food products	scaling up the process the lack of commercial applications	increase in unsaturated fat content reduction in saturated fat content lower mean serum triacylglycerol and FFA levels	Crisco—*trans* fat-free shortening Coasun—zero *trans*, low-saturate, oil-in-water structured emulsion, alternative to baking margarine. research studies: baking fats margarine and spreads meat products chocolate and chocolate pastes confectionary fillings ice cream yogurt cream cheese
direct dispersion	most commonly used process for creating oleogels one step process network formation with high oil binding capacity at much lower mass fraction of crystalline phase	low stability of the gel during storage due to the crystal aggregation		
oil binding method	porous cryogel could absorb oil at more than 100 times its own weight formation of strong gel	deformable gel non-self-standing gels		
emulsion template method	entrapment a large amount of liquid oil without showing any oil leakage over extended period of storage	limited dispersion of hydrophilic polymers in oil—ineffective in structuring oils lot of processing steps producing weak gels sensitive to homogenations, shearing, etc.		
bigel	thickening of water phase to add body to the emulsion while decreasing solid fat and/or total fat content of the product	rarely match the expected sensation by the consumer difficulty in controlling phase separation not thermo-reversible, not stable at higher temperatures		
bioengineered oleogels	obtaining microbial oils or organogelators by means of biotechnology valorization of food industry side streams and by-products sustainable technology	not studied enough low stability over storage period		
**Fat mimetics from nonlipid origin** **protein-based** **carbohydrate-based**	create creamy, smooth texture similar to fat stabilize water in the food product in a gel-like matrix provide creamy mouthfeel similar to that of full fat products	cannot be used in cooking oils or in products subject to frying conditions as the proteins are denatured and lose their functionality cannot provide all the functional and sensory advantages of conventional fats can be applied in low fat systems	digested and absorbed as protein/carbohydrate/dietary fiber low caloric value do not increase the risk of heart disease do not adversely affect blood lipid levels	Simplesse—fat replacer whey protein Dairy Lo—fat replacer whey protein K-Blazer—fat replacer protein N-Oil—maltodextrins fat replacer Maltrin M040—hydrolyzed starch fat replacer Paselli SA−2—enzyme modified potato starch fat replacer Oatrim—carbohydrate-based fat replacer Z-Trim—carbohydrate-based fat replacer

## Abbreviations

FFA—free fatty acid, TAG—triacylglycerol, MAG—monoacylglycerol, DAG—diacylglycerol, LMOG—low molecular weight oil gelators, HMOG—high molecular weight oil gelators, SFC—solid fat content, LDL—low-density lipoprotein, CMC—carboxymethylcellulose, SFAD—soybean fatty acid distillate, CBE—cocoa butter equivalent and CBI—cocoa butter improver.
